# Preventing the recurrence of acute anorectal abscesses utilizing a loose seton: a pilot study

**DOI:** 10.11604/pamj.2020.35.18.21029

**Published:** 2020-01-23

**Authors:** Timucin Erol, Bulent Mentes, Hakan Bayri, Igbal Osmanov, Sezai Leventoglu, Alp Yildiz, Mehmet Yorubulut, Ugur Sungurtekin

**Affiliations:** 1 Department of Surgery/Proctology, Memorial Ankara Hospital, Ankara, Turkey; 2 Department of Surgery, Gazi University Medical School, Ankara, Turkey; 3 Department of Surgery, Yildirim Beyazit University, Yenimahalle Research and Training Hospital, Ankara, Turkey; 4 Department of Radiology, Acibadem Hospital, Ankara, Turkey; 5 Department of Surgery, Pamukkale University Medical School, Denizli, Turkey

**Keywords:** Anorectal abscess, fistula-in-ano, seton

## Abstract

**Introduction:**

This pilot study aimed to document our results of treating anorectal abscesses with drainage plus loose seton for possible coexisting high fistulas or drainage plus fistulotomy for low tracts at the same operation.

**Methods:**

Drainage plus fistulotomy were performed only in cases with subcutaneous mucosa, intersphincteric, or apparently low transsphincteric fistula tracts. For all other cases with high transsphincteric fistula or those with questionable sphincter involvement, a loose seton was placed through the tract. Drainage only was carried out in 17 patients.

**Results:**

Twenty-three patients underwent drainage plus loose seton. Drainage plus fistulotomy were performed in four cases. None of the patients developed recurrent abscess during a follow-up of 12 months. Not surprisingly, the incontinence scores were similar pre and post-operatively (p=0.564). Only minor complications occurred in 4 cases (14.8 percent). Secondary interventions following loose seton were carried out in 13 patients (48.1 percent). At 12 months, drainage only was followed by 10 recurrences (58.8 percent; p<0.0001, compared with concomitant surgery).

**Conclusion:**

Concomitant loose seton treatment of high fistula tracts associated with anorectal abscess prevents abscess recurrence without significant complications or disturbance of continence. Concomitant fistulotomy for associated low fistulas also aids in the same clinical outcome. Concomitant fistula treatment with the loose seton may suffice in treating the whole disease process in selected cases. Even in patients with high fistula tracts, the loose seton makes fistula surgery simpler with a mature tract. Abscess recurrence is high after drainage only.

## Introduction

Concurrent definitive treatment of underlying fistulas at the time when the anorectal abscesses are drained is a controversial issue. Fistula-in-ano can increase the likelihood of abscess recurrence which then requires repeat drainage. Even with no recurrent abscess, further interventions may be needed for fistula-related symptoms such as discharge or perianal soreness. Regarding the pathogenetic association of the crypto-glandular abscess and fistula, attempting to eliminate the whole disease process concomitantly with the drainage of the abscess might be a reasonable approach mainly because i) the development of recurrent abscesses and repeated surgical drainages may be prevented and ii) eventual fistula surgery with additional anesthesia may be omitted. However, drawbacks exist based on the suggestions that i) some abscesses will not recur or evolve to become a fistula and ii) a combined procedure has a greater risk of anal incontinence. Nevertheless, the persistent entity of anorectal abscess and fistula is a common problem contributing significantly to the daily surgical workload, as well as expenses, day off work, and quality of life [1].

In this respect, there are growing inclination and efforts to treat any coexisting fistula tract concomitantly with the drainage of the abscess. Although about one-thirds of perianal abscesses were thought to be associated with fistula-in-ano [1-3], more recent studies aiming to detect any coexisting fistula tract have reported much higher rates (80-90 percent) of finding a fistula with a perianal abscess [4-6]. Two meta-analyses showed significant reductions in recurrence, persistent abscess/fistula or repeat surgery in favor of fistula surgery at the time of abscess incision and drainage (I&D) [7,8]. Even in children surgically treated for first-time perianal abscess, recurrence rates appear to be lowered by locating and treating coexisting fistulas [9]. Stratifying this group of patients, Oliver and coworkers recommended concomitant fistulotomy for subcutaneous, intersphincteric or low transsphincteric tracts, but not for high fistulas [6]. Likewise, the clinical practice guidelines of the American Society of Colon and Rectal Surgeons (ASCRS) have suggested that fistulotomy may be performed when a simple fistula is encountered during I&D [10]. This study aimed to document our results of treating anorectal abscesses with drainage plus loose seton for possible coexisting high or suspect fistula tracts or drainage plus fistulotomy for low fistulas at the same operation. Our rationale was to approach the surgical treatment of choice in anorectal abscesses which would offer the lowest recurrence without affecting continence.

## Methods

**Patients:** during a 12-month period starting in April 2016, 44 adult patients with primary or recurrent anorectal abscess, who fulfilled the inclusion criteria, were analyzed and documented on previously designed, standard forms. Twenty-nine of them applied to the Proctology Clinic and they were treated by or under the supervision of the dedicated proctologists, aiming to treat any coexisting fistula tract, as well. During the same time period, 15 patients who applied to the emergency unit on weekends or vacations were treated by the surgical registrars only by abscess drainage. Whenever possible, an urgent pelvic MRI was also performed and evaluated together with the dedicated radiologist, especially for clues for a coexisting fistulous tract. Patients with supralevator or horseshoe abscesses, diabetes or other endocrine disorders, allergy to latex, and those previously diagnosed as having Crohn’s disease, ulcerative colitis, or colorectal cancer were excluded. Informed consent was obtained from all patients.

**Figure 1:**
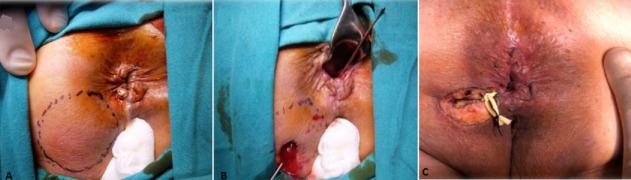
Left anterior abscess; (A) note purulent drainage from the internal opening; (B) drainage plus identification of the fistula; (C) conversion to hybrid seton at 12 months

**Definitions and surgery:** the primary endpoint was abscess recurrence. The secondary endpoints were incontinence scores and any surgical complications. Any abscess developing twice in the same quadrant was considered recurrent. A fistula was considered high transsphincteric if the track involved more than 30-40% of the thickness of the external anal sphincter. Almost all patients underwent surgery under spinal/epidural anesthesia and in prone jackknife position. Antibiotics were used selectively for patients with anorectal abscess complicated by cellulitis, induration, or overt signs of systemic sepsis, as well as those described by the American Heart Association [11]. Surgical treatment of the abscess consisted of incision with monopolar diathermy at the point of maximum fluctuation followed by circular excision of the skin, abscess debridement, and search for possible compartments. Before incision and drainage, an attempt was made to locate the internal opening, which could occasionally be identified when, under slight pressure above the abscess, purulent material was found oozing from a corresponding crypt (Figure 1). However, it may be difficult to determine the internal opening due to the presence of edema and obliteration by inflammatory debris. Any coexisting fistula tract was further searched with the help of the MRI findings, following Goodsall’s rule (fistulous abscesses in the posterior half generally follow a curved course towards the posterior midline, whereas those with an external opening in the anterior half follow a radial course) and/or by gentle probing, avoiding forceful maneuvers (Figure 1). Drainage plus fistulotomy were performed only in cases with subcutaneous-mucosa, intersphincteric or apparently low transsphincteric fistula tracks. For all other cases with high transsphincteric fistulae or those with questionable sphincter involvement, a single-strand, relatively thin, soft and elastic loose seton was placed through the tract (Figure 1). This seton was created by cutting a thin (2-3mm) circular strip from a surgical glove (latex surgical glove, Beybi™, Beybi Plastic Factory, Istanbul, Turkey), including its thicker sleeve.

**Follow-up:** postoperatively, the patients were re-examined at 7 and 28 days, 3, 6 and 12 months. They were encouraged to contact whenever they suspected a problem. The analysis aimed at one-year’s follow-up. At 6-month´s and/or 12-month’s follow-up, the patients with loose setons were informed about the possibility of eliminating the seton by various methods depending on the final topographic features of the tract. The technique and possible complications of removing the seton, fistulotomy, conversion to hybrid (elastic cutting) seton, or ligation of intersphincteric fistula tract (LIFT) were discussed in detail. Of the patients who underwent loose seton treatment, only those who kept their seton for 12 months were analyzed. After definitive fistula surgery or removal of the loose seton at 12 months, the Wexner incontinence scores and further complications were recorded again at 6 months [12].

**Statistical analysis:** SPSS software v23.0 (IBM Inc., Armonk, New York, USA) was used. Data were defined as the mean ± standard deviation. Chi-square test was used for the comparison of the independent variables and Wilcoxon test for the dependent groups. A p-value <0.05 was considered significant.

## Results

**Figure 2:**
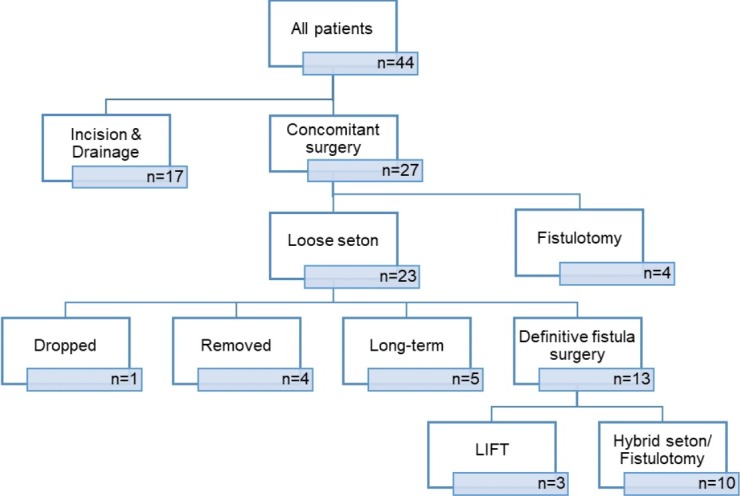
The detailed distribution of the patients treated for anorectal abscess

A total of 44 patients treated for anorectal abscess (33 males, mean age 39.1 ± 10.3) were followed for 12 months. Twenty-seven (20 males, mean age 39.3 ± 9.3) underwent concomitant surgery (CS) for co-existing fistula tract simultaneously with abscess drainage. In only two patients (6.9 percent) assigned to drainage with fistula treatment, the internal opening couldn´t be identified, and these patients were regarded as I&D only. The detailed distribution of the patients is outlined in Figure 2. Of the 27 CS patients, 17 had had previous abscess drainages and/or fistula. Four (14.8 percent) had low fistula tracts. Two of them had developed following lateral internal sphincterotomy and all four were treated with fistulotomy. Twenty-three patients underwent drainage plus loose seton. Five patients (18.5 percent) kept their loose seton for longer than 12 months. One was diagnosed to have Crohn’s postoperatively, two were anterior transsphincteric fistula in women and two patients simply chose to proceed with their seton. The seton was removed in another four patients (14.8 percent) at 12-month’s follow-up. It dropped accidentally in a single patient at 5 months. Definitive fistula surgery was carried out in 13 patients (48.1 percent) 12-month’s follow-up.

**Figure 3:**
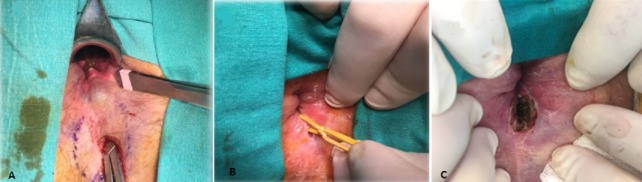
(A) drainage of the anorectal abscess with identification of the internal opening; (B) mature fistula tract became superficial at 12 months; (C) lay-open of the resultant simple fistula

**Figure 4:**
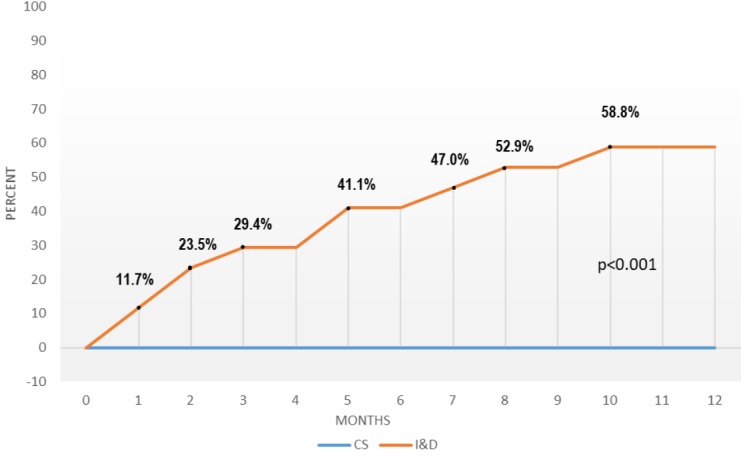
The recurrence rates of the concomitant surgery and incision & drainage groups

Ten underwent fistulotomy or conversion to a hybrid seton, as described previously, because the tract had matured to a safe and superficial topography [13] (Figure 3). Three patients (11.1 percent) underwent LIFT because of high fistula tracts. None of the patients in the CS group developed recurrent abscess during a follow-up of 12 months. Not surprisingly, the CCIS scores were identical in 21 patients at 12 months (0.29 ± 0.86 vs. 0.33 ± 0.83; p=0.564). However, 6 months following definitive treatment, a statistically significant difference occurred (0.29 ± 0.86 vs. 0.63 ± 0.96; p<0.0001). Only minor complications occurred (4 cases-14.8 percent of urinary retention and one arrhythmia). One patient, who eventually underwent hybrid seton treatment at 12 months, and the patient who accidentally lost the seton developed fistulas (resulting in a final healing rate of 92.6 percent). A single patient who underwent secondary fistulotomy required hemostasis for bleeding with electrocautery. Incision and drainage were carried out in a total of 17 patients. Ten patients had had previous abscess drainages and/or fistula. The demographics of the two groups were similar. In a year, 10 recurrences (58.8 percent) were noted. Three patients (17.6 percent) had fistulas without any intercurrent abscess, and only 4 healed completely (23.5 percent). It´s noteworthy that all these healed cases had experienced an abscess for the first time. This recurrence rate was significantly higher than that of the CS group p<0.0001 (Figure 4). Although most of these recurrences were treated by concomitant fistula surgery, they were not reinstated further in the study.

## Discussion

The results of our study have suggested that identification and loose seton treatment of high fistula tracts associated with anorectal abscess prevents abscess recurrence without significant complications or disturbance of continence. Concomitant fistulotomy for associated low fistulas also aids in the same clinical outcome. A fistula tract was identified in 93.1 percent of the cases. Although a draining seton has been suggested to be a safe and acceptable treatment as an alternative to ‘primary’ fistulotomy, our study is probably the first to present data in this setting [10]. The loose seton prevented abscess recurrences in all patient within the time limits. As expected, no specific complications or anal incontinence occurred. The literature and guidelines have been rather reluctant to suggest definitive fistula surgery together with abscess drainage [10,14]. The fact that concomitant fistula treatment has largely been limited to fistulotomy or cutting seton has contributed negatively to the development of this reasonable strategy. Although Read mentioned performing a primary fistulotomy for all their perianal abscesses if a fistula was found concomitantly, Hebjorn did the first controlled trial [2,4]. Their technique was not exactly synchronous drainage plus fistulotomy and the 40 percent rate of minor continence problems reported after CS put shade on a more liberal approach for many years. Oliver and coworkers randomized a large group of patients [6]; however, more than two-thirds of their patients surprisingly had subcutaneous or intersphincteric abscesses. This patient distribution is different to our findings and knowledge that anorectal abscesses are more common in the perianal and ischiorectal spaces, and less common in the intersphincteric, supralevator, and submucosal locations [2,15].

A major problem with CS is the fact that anorectal abscesses are defined by the anatomic space in which they develop while the topographic features of a fistula are defined considering sphincter involvement [2,15]. Our strategy of treating any associated suspect fistula tract with the loose seton appears to solve this possible misinterpretation problem considering sphincter involvement, as well as the related and feared possibility of disturbance of continence. Even if the patient eventually turns out to harbor Crohn’s disease, the strategy is valid. In addition to preventing abscess recurrence, concomitant fistula treatment with the loose seton may suffice in treating the whole disease process in selected cases. Removal of the seton in four patients (15 percent) at 12 months due to their refusal for further surgery resulted in healing within the time limits. Although this strategy was supported by the recent study of Oluwatomilayo and coworkers, we are yet far from suggesting it as a standard procedure [16]. More important was the observation that after the inflammation resolved, the seton provided a mature and eventually more superficial tract in 37 percent of the patients. Simple lay-open or conversion to a hybrid seton was then possible on an outpatient basis. Even in patients with high fistula tracts, the operation was simpler with a mature tract. The loose seton converts a septic condition that may recur to an elective one that can be treated in tertiary centers. The surgical approach to fistula is a vast topic beyond the aim of this trial, and it may naturally affect continence. A control group was, by definition, not intended. However, 17 patients who underwent I&D only accumulated during the study. The recurrence rates as reported in the literature are almost exclusively based on data obtained from retrospective studies. Therefore, the exact number of recurrent abscesses and persistent fistulas after I&D is still unknown. We noted a 58.8 percent rate of abscess recurrence in a year and this rate will probably increase in long term.

## Conclusion

In conclusion, further controlled trials are inspired by the results of this study suggesting that: i) associated fistula tracts can be identified in the majority of cases with acute anorectal abscess; ii) loose seton treatment of high fistula tracts associated with anorectal abscess prevents abscess recurrence without complications or disturbance of continence. Concomitant fistulotomy for associated low fistulas also aids in similar clinical outcome; iii) concomitant fistula treatment with the loose seton may suffice in treating the whole disease process in selected cases; iv) after the inflammation resolves, the seton provides a mature and usually a more superficial tract. Therefore, an urgent, septic condition is converted to an elective one for tertiary centers; and v) abscess recurrence is high after I&D only.

### What is known about this topic

- Perianal abcess recurrence is possible due to untreated fistula if only simple drainage performed;
- Combined surgery for the treatment of abcess and fistula at the same operation can cause fecal incontinence.

### What this study adds

- Utilising a loose seton can prevent recurrence of abcess and avoids or fascilitates the fistulae surgery during follow up without major complications.
